# Hydrothermal treatment of Ti surface to enhance the formation of low crystalline hydroxyl carbonate apatite

**DOI:** 10.1186/s40824-014-0022-y

**Published:** 2015-01-20

**Authors:** Soyoung Yang, Sujeong Lee, Indu Bajpai, Sukyoung Kim

**Affiliations:** Materials Science and Engineering, Yeungnam University, 280 Daehak-Ro, Gyeongsan, Gyeongbuk 712-749 South Korea

**Keywords:** Titanium, Hydrothermal treatment, NaOH-etching, SBF test, Ca-P coating

## Abstract

**Background:**

Ti and its alloys have been widely used as orthopedic and dental implants due to their outstanding mechanical properties and biocompatibility. However, long time is required to form bond between Ti implant and surrounding tissues. Therefore, these implants necessitate surface treatment such as mechanical/chemical treatment and coating of bioactive materials for improving the osseointegration.

**Results:**

This study was focused on the calcium-phosphate (Ca-P) coating on machined Ti, blasted-Ti (B-Ti), and blasted-NaOH-etched-Ti (BNH) surfaces by hydrothermal method to evaluate the ability of HA formation. Nanostructured morphology was created by NaOH etching on blasted-Ti surface. XRD analysis confirmed the existence of sodium titanate phase on such samples. Rutile and anatase phases along with hydroxyapatite were observed after hydrothermal treatment in Ca-P solution. Substantial hydroxyapatite together with TiO_2_ was observed during hydrothermal treatment at 200°C for 12 hrs. Blasted-NaOH-etched samples (BNH-Ti) revealed appreciable bone-like apatite formation as compared to machined-Ti and blasted-Ti (B-Ti) surfaces. However, maximum HA formation was confirmed on Ca-P coated-BNH samples (BNHA-Ti-200-12) by XRD and ICP analysis.

**Conclusion:**

Multistep surface treatment adopted in current study would be effective to enhance HA formation on Ti surface.

## Background

Although, durability of Titanium (Ti) implants has been already proven in biomedical field, slow as well as poor implant-bone interface are still a big challenge. To resolve this issue, various surface modifications using physical, chemical methods and coating with bioactive material were used to promote early osseointegration and fixation [1,2]. Surface modification by physical treatment such as girt-blasting and machining improved the biomechanical fixation between implant and human body [3]. Bioactive surfaces were achieved by chemical treatment with acid/alkali solution. Especially, Sodium titanate subjected to soaking in SBF solution and subsequent heat-treatment formed bone like apatite-layer [4]. In addition, some researcher groups have studied about coating method such as plasma spraying, sol–gel, electron beam sputtering with bioactive materials. Plasma spray coating has been widely utilized with hydroxyapatite (HA) and bioglass. Although, owing advantage in terms of obtaining high crystallinity on surface, it was found to be difficult to control the thickness and week adhesion at the coating-substrate interface [5]. Sol–gel and dip-coating with HA, bioglass and water glass were reported to be easy method of coating on implant surface [6]. Park et. al reported that coating using water-glass sol was effective for apatite layer formation and cell activation. However these kinds of physical coating methods exhibited week adhesion strength on metal surface [7].

On the other hands, hydrothermal method seemed to be relatively simple and effective due to chemical reaction as well as coating. Although to be stable materials at an ambient temperature, the chemical reaction of those could be promoted under the condition of hydrothermal treatment. Hamade et al. conducted surface coating with hydrothermal treatment using CaO solution. They reported that hydrothermal treatment in CaO solution enhanced the precipitation of apatite on the titanium surface [8]. Ishikawa et al. proposed that Ti-O-Ca boding formed by hydrothermal treatment in CaCl_2_ solution was effective for the fabrication of titanium implant with good bioactivity and osteoconductivity [9,10]. Hu et al. referred to a one-step hydrothermal process with hydroxyapatite (HA) suspension to coat Ti surface [11].

In this study, multiple surface treatment processes such as grit-blasting, NaOH etching and hydrothermal treatment on the Ti surfaces were conducted to improve the bone-like apatite formation and early osseonintegration. Especially, this study was focused on the calcium phosphate coating by hydrothermal method on various types of surface such as Ti, blasted Ti (B-Ti), and blasted-NaOH etched Ti (BNH-Ti) and evaluation of apatite forming ability on these surfaces in SBF environment. The appropriate treatment conditions for the bioactive coating were investigated in terms of temperature and time of hydrothermal treatment. Calcium phosphate coating was conducted by hydrothermal treatment at 120, 160, 200°C for 3, 6, 12, 24 hrs. Surface chemistry and morphology of calcium phosphate coated Ti surface were characterized using SEM and XRD. The ability of forming bone-like apatite layer was studied in SBF.

## Methods

### Preparation of the samples

Firstly, commercially pure Ti discs (cp-Ti, grade 4) with 12 mm diameter and 2 mm thickness were polished using SiC papers (Daesung, Korea). Then polished discs were blasted using 250–300 HA grits and then washed with 1 vol% HCl solution in an ultrasonic bath for 2 mins to remove HA residues on the surfaces. These discs were again cleaned in an ultrasonic bath with pure water and ethanol to remove the HCl residues from the Ti discs. Lastly, washed Ti discs were soaked into a 5 N NaOH solution at 60°C for 24 hrs, followed by subsequent heat treatment at 600°C for 1 h.

### Hydrothermal treatment

Calcium phosphate (Ca-P) solution was prepared by mixing 20 mM of calcium nitrate tetrahydrate and 12 mM of disodium phosphate for 12 h on magnetic stirrer and pH was adjusted to 11 by titrating with NH_4_OH. Ti, B-Ti, BNH-Ti were fixed into the teflon jig and prepared calcium phosphate solution was filled in a teflon vessel. The vessel was heated at 120, 160 and 200°C for 3, 6, 12 and 24 h. Furthermore, calcium phosphate coated Ti substrates was rinsed with pure water for several times and dried in oven at 60°C.

### Biomineralization

Surface treated Ti discs were soaked in a 40 ml of simulated body fluid (SBF) solution at 36.5°C, 80 rpm in a shaking incubator for 1, 7 days. SBF was prepared by dissolving NaCl, NaHCO_3_, KCl, K_2_HPO_4_ · 3H_2_O, MgCl_2_ · 6H_2_O, CaCl_2_, Na_2_SO_4_ and Tirs- (hydorxymethyl) aminomethane in ultra-pure water and titrated at pH 7.40 using 1 M HCl, which was nearly equal to ion concentrations (Na^+^ 142.0 mM/L, K^+^ 5.0 mM/L, Mg^2+^ 1.5 mM/L, Ca^2+^ 2.5 mM/L, Cl^−^ 103.0 mM/L, HCO_3_^−^ 4.2 mM/L, HPO_4_^2−^ 1.0 mM/L, SO_4_^2−^ 0.5 mM/L) in human blood plasma was used to estimate bioactivity [12].

### Characterization of the Ti surface

The modified-Ti surfaces were analyzed using thin-film X-ray diffraction (PANalytical, Netherlands) to identify the phases. The surface morphology and chemical composition of samples were observed under FE-SEM (field emission scanning electron microscopy, Hitachi, Japan) and EDS (energy dispersive X-ray spectroscopy, Hitachi, Japan). The weight changes of the specimens were examined before and after immersion in SBF solution by an electronic balance (4-digit accuracy balance, AND HR-120, Japan). Concentration of calcium and phosphorus ions remained in solution after SBF test was measured by ICP-AES (OPTIMA8300, Perkin Elmer, USA).

## Results and discussion

**Figure 1:**
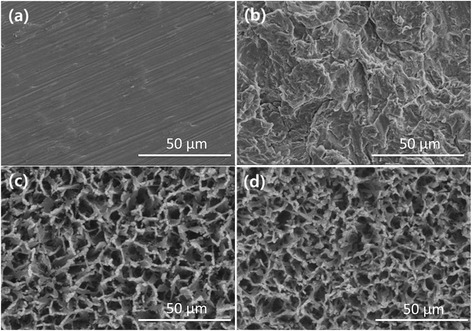
**Surface morphology of (a) Machined- (Ti), (b) blasted- (B-Ti), (c) NaOH etched- (N-Ti) and (d) blasted-NaOH etched Ti (BNH-Ti).**

Surface microstructure of machined titanium (Ti), blasted Ti with HA grits (B-Ti), chemically etched Ti with a sodium hydroxide solution (N-Ti) and blasted-NaOH etched Ti (BNH-Ti) were shown in Figure 1. Macro sized coarse surface (Figure 1b) was verified after blasting with HA whereas nano-sized porous network structure was confirmed after soaking in sodium hydroxide solution at 60°C for 1 day (Figure 1c). Surface topography of blasted -NaOH etched-Ti surface (Figure 1d) was observed to be composed of nano-sized network structure on the coarse surface.

**Figure 2:**
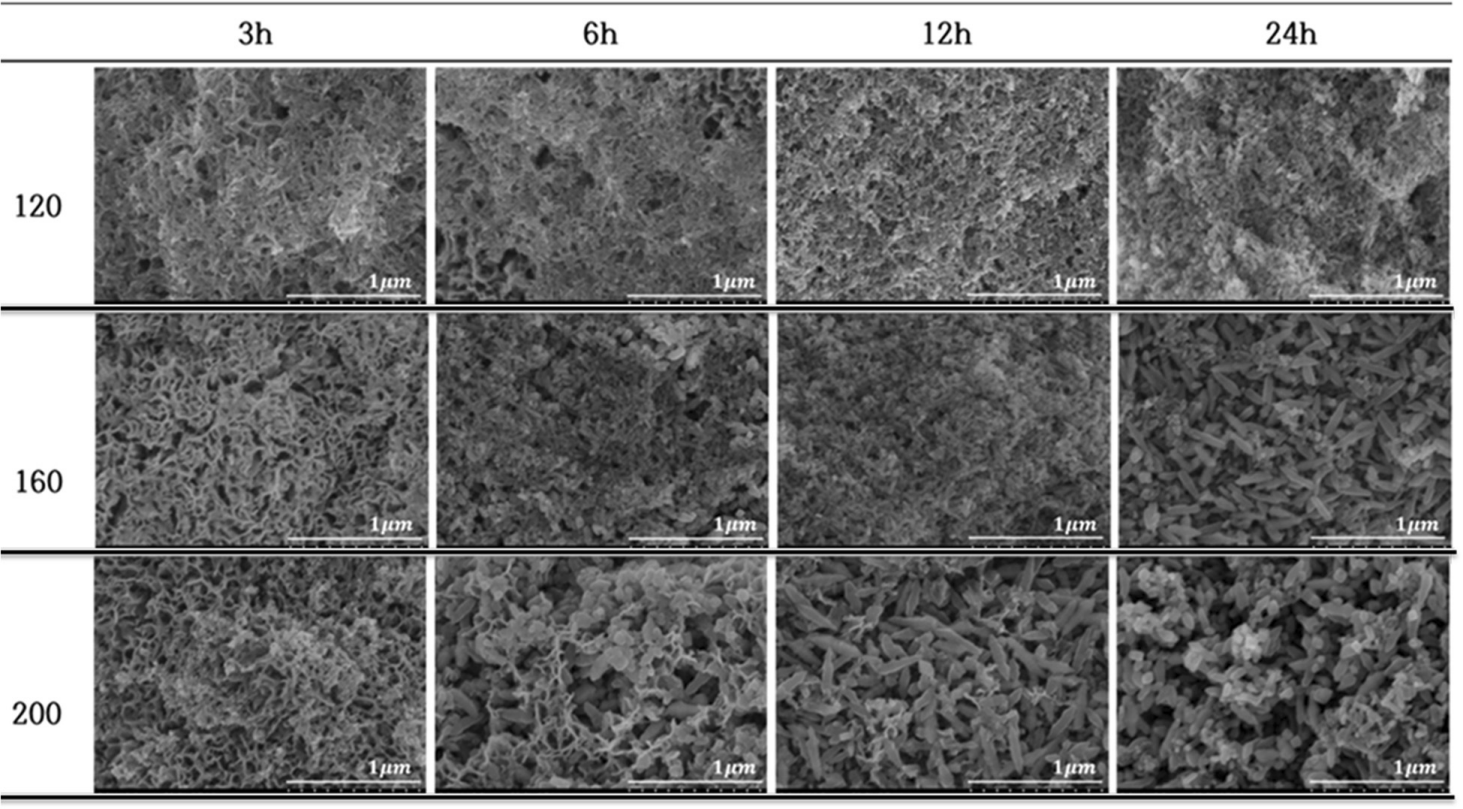
**SEM images of hydrothermally treated blasted-NaOH etched-Ti samples (BNHA-Ti) in calcium phosphate solution with pH10 at 120, 160 and 200°C** **for 3, 6, 12 and 24 hrs.**

**Figure 3:**
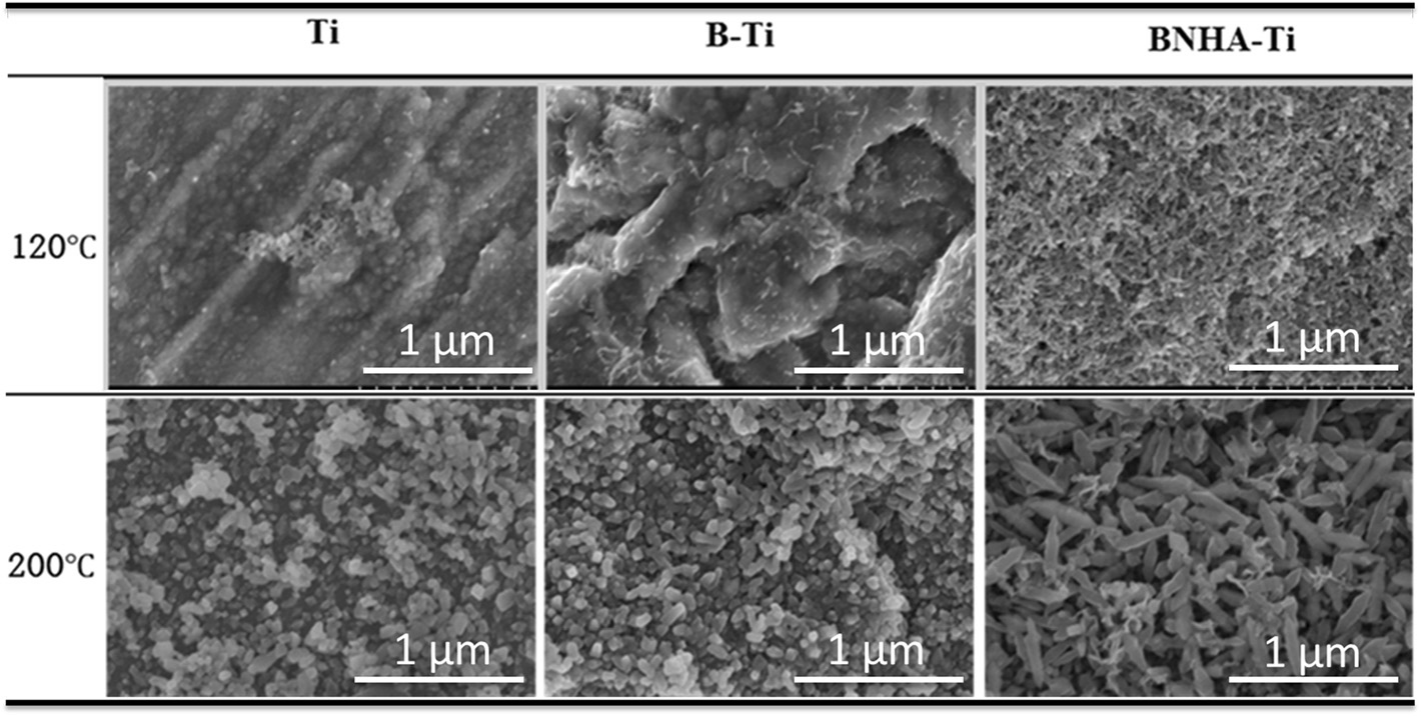
**SEM images of Ti, B-Ti, BNHA-Ti by hydrothermal treatment in calcium phosphate solution with pH 10 at 120 and 200°C** **for 12 hrs.**

Hydrothermally treated surfaces of BNH-Ti substrates (named as BNHA-Ti) at 120, 160, 200°C for 3, 6, 12, 24 hrs were shown in Figure 2. The rod-type of HA crystals appeared on the surface after hydrothermal treatment. The rod-type of anatase crystals as well as HA crystals were observed at higher temperature and in longer reaction time. Anatase crystals came out on the surface of titanium from inside after hydrothermal treatment at 200°C for 6 hrs. Therefore, mixed-structure was formed as HA and anatase after hydrothermal treatment at 200°C for 12 hrs. However, the anatase crystals predominated rather than HA by further heat-treatment. On the basis of such observations, Ti and B-Ti samples were hydrothermally treated at 120 and 200°C for 12 hrs in Ca-P solution (Figure 3) and anatas-TiO_2_ and HA crystals were observed at 200°C treatment temperature.

**Figure 4:**
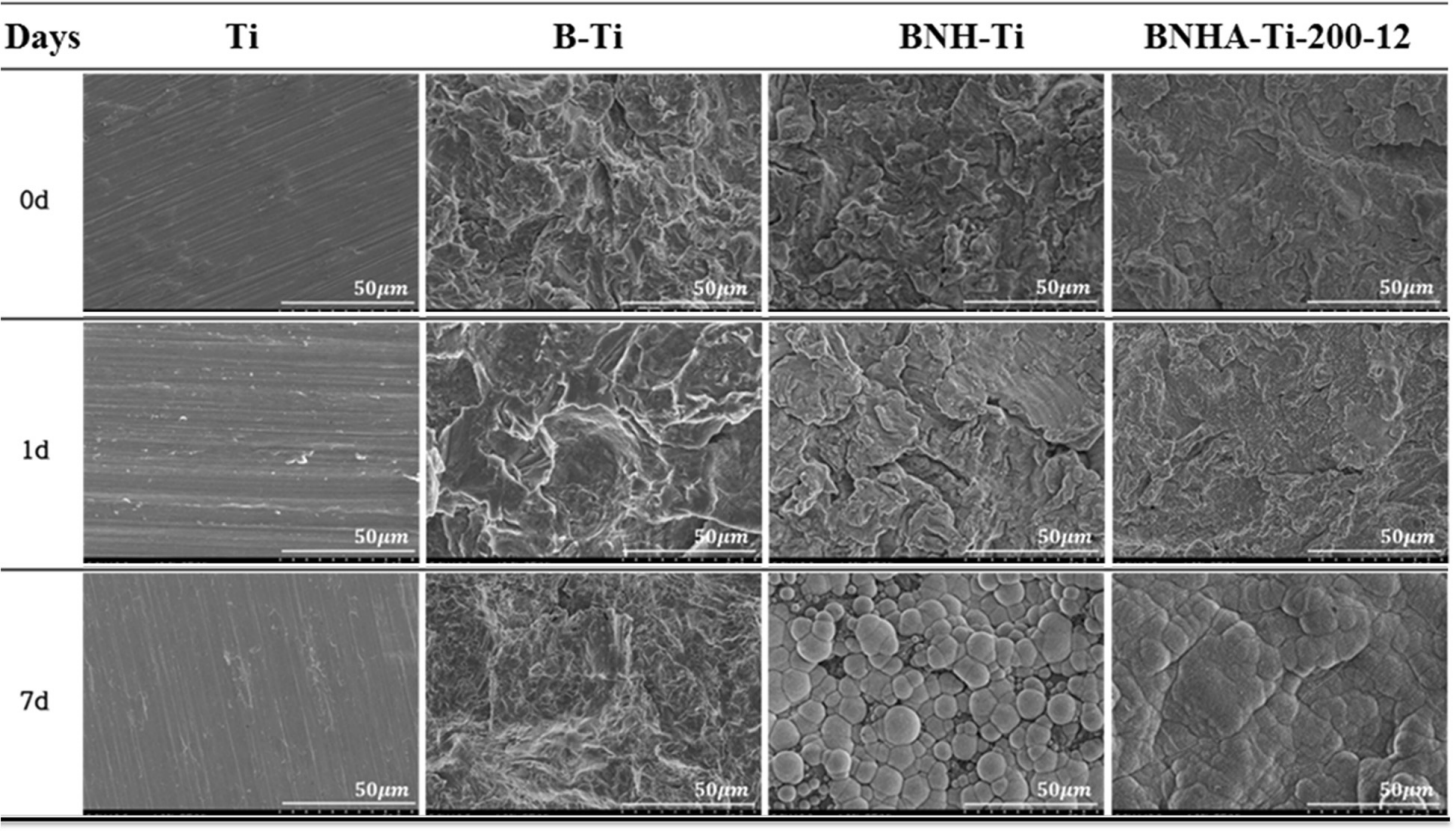
**SEM images of surfaces of Ti, B-Ti, BNH-Ti, BNHA-Ti-200-12 after soaking in SBF solution at 37°C** **for 0, 1 and 7 days.**

Various type of titanium surfaces were immersed into simulated body fluid (SBF) for 1 to 7 days to observe apatite formation activity. The surface micrographs of Ti, B-Ti, BNH-Ti, BNHA-Ti after soaking in SBF solution at 37°C were shown in Figure 4. Apatite layers did not appear on Ti and B-Ti surface after soaking in SBF solution for 1 and 7 days. Apatite nucleation was observed on BNH-Ti and BNHA-Ti surfaces after 1 day immersion in SBF, but it was significantly increased on BNHA-Ti surface after 7 days.

**Figure 5:**
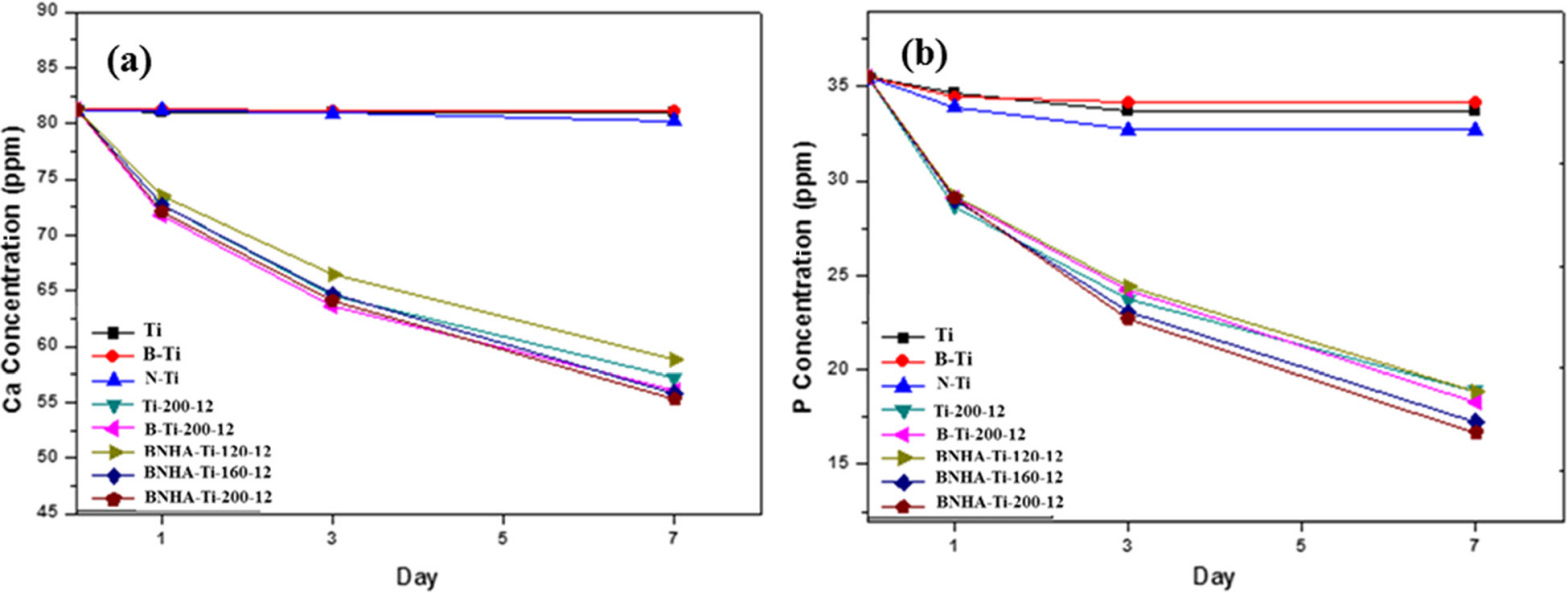
**Concentrations of remained Ca and P ions in SBF after 1, 3 and 7 day immersion of various types of titanium samples; machined, and blasted, alkali etched, blasted-etched.**

Figure 5 showed the ICP analysis of remained calcium and phosphorus ions concentration in SBF solution with immersion time of specimens. The concentration of calcium and phosphorus was not changed on mechanically treated Ti, B-Ti surfaces during immersion upto 7 days. On the other hand, in case of hydrothermally treated BNHA-Ti specimen, concentration of calcium and phosphorus was decreased with immersion time. Also, the concentrations of calcium and phosphorus which remained in SBF after removing hydrothermally treated specimens (Ti-200-12, B-Ti-200-12) were rapidly decreased. It was considered that the decrease of calcium and phosphorus concentrations in SBF was due to the precipitation of apatite on the titanium surface. Therefore it was expected to be effective for the formation of apatite on the hydrothermally treated surface than chemically treated. It was believed that hydrothermally treated surfaces were effective for nucleation of apatite than the others.

**Figure 6:**
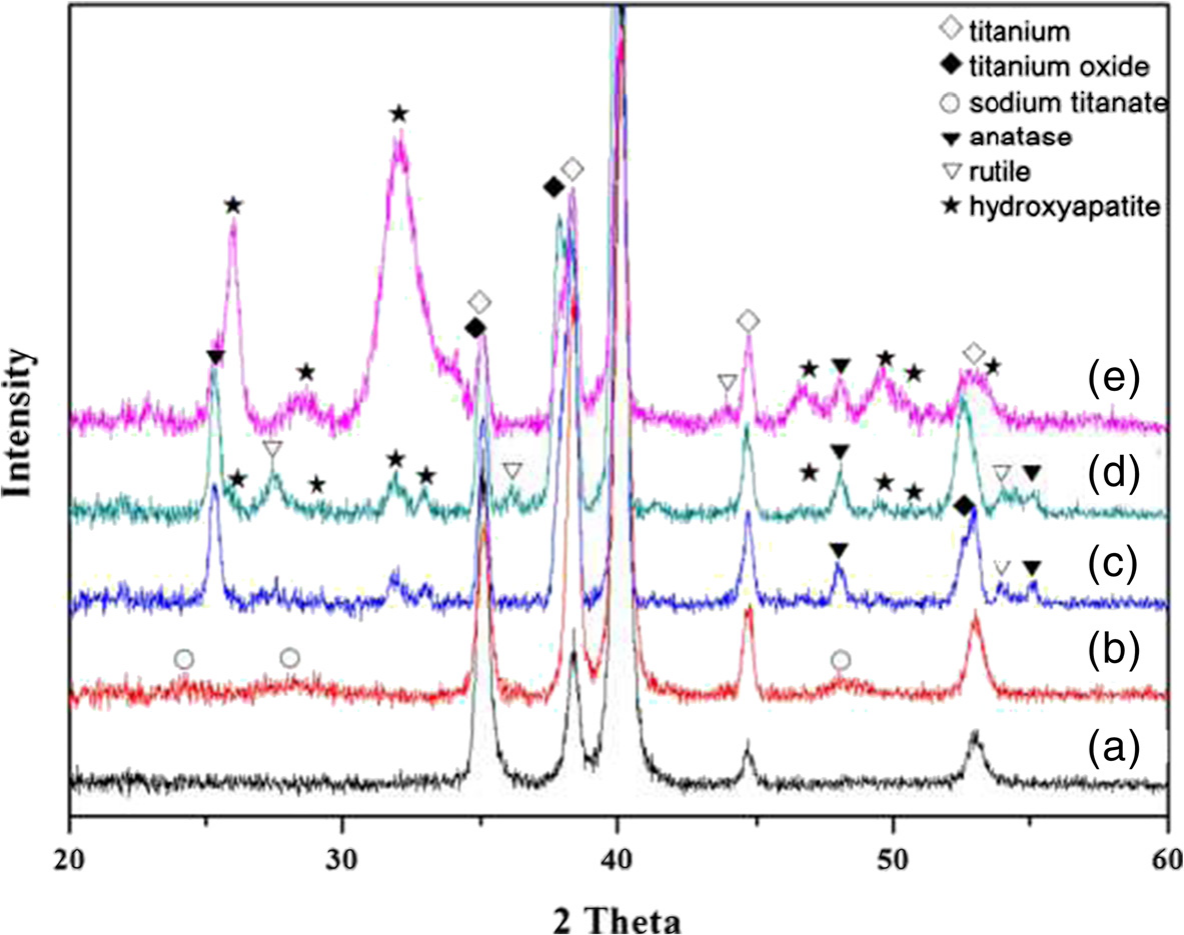
**XRD patterns of (a) machined-Ti, (b) BNH-Ti, (c) BNHA-Ti-200-12, (d) BNHA-Ti-200-12 immersed in SBF solution for 1 day, and (e) BNHA-Ti-200-12 immersed in SBF solution for 7 days.**

XRD analysis of Ti, BNH-Ti, BNHA-Ti-200-12 and BNHA-Ti-200-12 after SBF test for 1, and 7 days were shown in Figure 6. Sodium titanate appeared on the chemically treated surface with NaOH solution (BNH-Ti). It confirmed HA phase after coating with calcium phosphate suspension by hydrothermal method. Although, one day immersion of BNHA-Ti-200-12 samples in SBF slightly increased HA precipitation on the surface, significant increase in intensity corresponding to HA was observed after 7 days. It was found that calcium phosphate layer coated after several heat treatments was highly effective for the formation of the apatite in simulated body fluid.

As it was evident from the results that NaOH etched Ti surface (BNH-Ti) showed significantly better apatite formation ability than the machined-Ti and blasted-Ti surfaces. The reason behind this is that sodium and oxygen ions entered into the Ti surface during the reaction, which led to formation of sodium titanate and titanium dioxide [4]; both phases were clearly identified by XRD analysis of BNH sample (Figure 6). When these etched samples were exposed in SBF solution, sodium ions were exchanged with H_3_O^+^ in SBF and formed Ti-OH bond whereas released sodium ions increased local pH of the sample surface that activated the apatite precipitation [4]. When NaOH-etched-Ti samples were hydrothermally treated in Ca-P solution, sodium ions were released in the reaction solution and positively charged Ca^+2^ ions accumulated in the groves of the surface and reacted with negatively charged PO_4_^−3^ ions of the solution to form a stable phase as crystalline hydroxyapatite. XRD analysis (Figure 6) and microstructure (Figure 2) of BNH200-12 samples showed the existence of TiO_2_ rods and hydroxyapatite. Subsequently, when BNHA-Ti-200-12 samples were exposed to SBF solution, inherently present hydroxyapatite on the surface worked as nucleation sites and stimulated the bone-like apatite formation on the surface. As indicated by the ICP analysis of the SBF solution, all samples hydrothermally treated in Ca-P solution showed better apatite precipitation than the machined Ti and blasted Ti. In addition, among all Ti surfaces, maximum as well as fastest bone-like apatite precipitation was observed on multistep treated Ti samples (BNHA-Ti-200-12) as indicated by highest intensity corresponding to HA peak in Figure 6 as well as ICP analysis (Figure 5), where the maximum loss of calcium and phosphorous ions from SBF solution indicated the highest carbonate HA formation.

## Conclusions

In the current work, multiple ways of surface treatments as blasting, alkali etching and Ca-P coating were carried out to improve bioactivity of Ti surface. Particularly, sodium titanate was observed on blasted-Ti surface after etching in NaOH solution. Hydroxyapatite crystals were formed easily on titanium surface during hydrothermal treatment. Moreover, HA layer on the surface was enhanced with increasing temperature and time. Though, manifest HA layer along with analase was formed on all of samples (Ti, B-Ti, BNH-Ti) after hydrothermal treatment at high temperature (200°C) for 12 hrs. However, anatase was found to be dominant than HA as further hydrothermal treatment (for 24 hrs) inhibited formation of HA on the surface. For this reason, the proper temperature and time were important to form HA during hydrothermal treatment. It was confirmed that the hydrothermally treated surface was effective to form bone-like apatite layer in SBF test. Maximum carbonate HA on Ti surface was found at the multistep treated Ti surface (BNHA-Ti-200-12). In conclusion, hydrothermal method with calcium phosphate suspension was found to be simple and effective method for HA coating on Ti implants.

## Availability of supporting data

The data sets supporting the results of this article are included within the article.
